# Comparison of HIF-1α and survivin levels in patients with diabetes and retinopathy of varying severity

**DOI:** 10.5935/0004-2749.2023-0112

**Published:** 2024-03-27

**Authors:** Burak Bilgin, Semsettin Bilak, Yusuf Özay

**Affiliations:** 1 Department of Ophthalmology, Faculty of Medicine, Kahramanmaras Sutcu Imam University, Kahramanmaras, Turkey; 2 Department of Ophthalmology, Faculty of Medicine, Adıyaman University, Adıyaman, Turkey; 3 Department of Medical Biology, Faculty of Medicine, Adıyaman University, Adıyaman, Turkey

**Keywords:** Survivin, HIF-1, Diabetic retinopathy, Hypoxia, Neovascularization

## Abstract

**Purpose:**

This study measured serum hypoxia--inducible factor-1 (HIF-1α) and survivin levels in patients with diabetes and investigated their association with the severity of retinopathy.

**Methods:**

This study included 88 patients with type 2 diabetes mellitus who underwent routine eye examinations. Three groups were created. Group 1 consisted of patients without diabetic retinopathy. Group 2 included patients with non-proliferative diabetic retinopathy. Group 3 included patients with proliferative diabetic retinopathy. To measure serum HIF-1α and survivin levels, venous blood samples were collected from patients.

**Results:**

The mean HIF-1α levels in groups 1, 2, and 3 were 17.30 ± 2.19, 17.79 ± 2.34, and 14.19 ± 2.94 pg/ml, respectively. Significant differences were detected between groups 1 and 3 (p=0.01) and between groups 2 and 3 (p=0.01). The mean survivin levels in groups 1, 2, and 3 were 42.65 ± 5.37, 54.92 ± 5.55, and 37.46 ± 8.09 pg/ml, respectively. A significant difference was only detected between groups 2 and 3 (p=0.002).

**Conclusion:**

The present study revealed that serum HIF-1α and survivin levels are increased in patients with non-proliferative diabetic retinopathy compared to those in patients without diabetic retinopathy.

## INTRODUCTION

Diabetes mellitus (DM) is a chronic disease that destroys the body by causing macrovascular and microvascular complications. Diabetic retinopathy (DR) is the most common microvascular complication of DM, and it results in vision loss. Vascular changes in DR have been explained by several biochemical, hemodynamic, and immunological mechanisms^(1^-^3)^. DR is diagnosed via clinical fundus examination of vascular abnormalities in the retina. Clinically, DR is classified as non-proliferative or proliferative^(4)^. Non-proliferative DR is a milder form that represents an earlier stage of DR. Proliferative DR is characterized by neovascularization, and represents an advanced stage of DR^(5)^. An ischemic process begins long before the appearance of visible anatomical changes, in which many trophic factors, complement factors, cytokines, and mediators are involved. The ischemic process initiated by hypoxia appears to be the main contributor to ocular complications, which cause retinal damage^(6)^.

Hypoxia occurs when vascular blood supply is impaired and tissue oxygen levels are lower than demanded. Under hypoxic conditions, hypoxia-inducible factor-1 (HIF-1) acts as a transcriptional regulator of several critical genes for cellular functions. HIF-1 was first detected in the kidneys and liver^(7)^. HIF-1 is composed of two subunits, namely HIF-1α and HIF-1β^(8)^. HIF-1α is the main active subunit, and its expression triggers the activation of vascular endothelial growth factor (VEGF) and other angiogenic factors, leading to angiogenesis^(9)^.

Survivin is a member of the inhibitor of apoptosis protein family that plays a key role in cellular proliferation and the inhibition of apoptosis^(10)^. Previous studies revealed that survivin is highly expressed in fetal tissues and malignant tumors but is rarely detectable in most healthy adult tissues^(11)^. Apoptosis is critical in the process of vascular remodeling. Survivin has a unique ability to enhance angiogenesis by inhibiting endothelial cell apoptosis^(12)^. Recent studies illustrated that survivin expression increases in response to conditions associated with vascular damage, including diabetic vasculopathy^(13)^.

Biomarkers are valuable and useful molecules found in blood, vitreous humor, aqueous humor, or other body fluids that can provide insights into the risks of diseases, thereby facilitating patient identification and treatment management^(14^,^15)^. In the literature, studies described alterations of various biomarker levels in body fluids such as vitreous humor, aqueous humor, tears, and serum in patients with diabetes^(16^-^18)^.

In this study, we measured HIF-1α and survivin levels in the serum of patients with diabetes as potential biomarkers and investigated their potential relationships with the severity of retinopathy.

## METHODS

This study was performed in the Adıyaman Unıversity School of Medicine Ophthalmology Department, and it included 88 patients with type 2 DM who visited our clinic between July 2021 and December 2021. All procedures followed the guidelines of the Declaration of Helsinki, and written informed consent was obtained from all patients. Local ethics committee approval was obtained (Ethics Committee for Clinical Trials of Adıyaman University, 2021/05-19). Patients who underwent follow-up after a diagnosis of type 2 DM and routine eye examinations were included in the study. Three groups were created. Group 1 consisted of patients without DR. Group 2 consisted of patients with non-proliferative DR. Group 3 consisted of patients with proliferative DR. A complete ophthalmic examination, including an assessment of best corrected visual acuity (BCVA), slit-lamp biomicroscopy, and fundoscopy, was performed in all patients. The diagnosis of DR was based on the biomicroscopic fundus examination. Patients with autoimmune (rheumatologic) diseases, systemic hypertension, ocular trauma, ocular inflammation featuring uveitis, and type 1 DM were excluded from the study. Patient files were scanned, and information such as age, gender, and the results of routine laboratory tests was obtained retrospectively.

### Enzyme-linked immunosorbent assay (ELISA)

To measure serum HIF-1α and survivin levels, approximately 5-6 ml of venous blood were collected from patients using serum-separating tubes, and serum was obtained via centrifugation at 2500 rpm for 15 min. Subsequently, samples were maintained at -86°C until protein measurements. HIF-1α and survivin levels were measured using commercially available ELISA kits (Sunred Biological Technology, Shanghai, China) in accordance with the manufacturer’s protocol. Briefly, blank, standard, and sample wells were created, and reagents were prepared and incubated at room temperature for 30 min. Following incubation, samples, standards, and reagents were loaded, gently mixed, and incubated for 60 min at 37°C to permit reaction. Then, samples were discarded, and plates were rinsed five times with washing buffer. Following washing, the wells were incubated with chromogen A and B solutions for 10 min at 37°C. Subsequently, the reaction was terminated using stop solution, and absorbance was measured at 450 nm using the EZ Read 400 instrument (Biochrom, Cambridge, UK). A standard curve was plotted using a four-parameter logistic regression model to determine actual protein concentrations.

### Statistical analysis

Statistical analysis was performed using Statistical Package for Social Sciences 21.0 for Windows (IBM, Armonk, NY, USA). Frequency, percentage, and mean ± standard deviation (SD) were used as descriptive statistics. The Shapiro-Wilk test was used to determine whether the data followed a normal distribution, which was indicated by p>0.05. One-way ANOVA was used to compare the means of normally distributed data, and the Kruskal-Wallis test was used for non-normally distributed data. P<0.05 indicated statistical significance.

## RESULTS

The sex distribution (p=0.894) and mean age (p=0.552) were comparable among the groups. The mean HIF-1α levels in groups 1, 2, and 3 were 17.30 ± 2.19, 17.79 ± 2.34, and 14.19 ± 2.94 pg/ml, respectively. HIF-1α levels significantly differed among the groups (p=0.04). In the sub-group analysis, HIF-1α levels significantly differed between groups 1 and 3 (p=0.01) and between groups 2 and 3 (p=0.01) but not between groups 1 and 2 (p>0.99). The mean survivin levels in groups 1, 2, and 3 were 42.65 ± 5.37, 54.92 ± 5.55, and 37.46 ± 8.09 pg/ml, respectively, revealing a significant difference among the groups (p=0.03). In the sub-group analysis, survivin levels only differed between groups 2 and 3 (p=0.002). The demographic characteristics of the patients and results are presented in table 1.

**Table 1:** The demographic characteristics of the patients and comparisons of HIF-1α and survivin levels among the groups

	Group 1	Group 2	Group 3	p	p between groups 1 and 2	p between groups 1 and 3	p between groups 2 and 3
**HIF-1α** (pg/ml)	17.30 ± 2.19	17.79 ± 2.34	14.19 ± 2.94	0.04	1.00	0.01	0.01
**Survivin** (pg/ml)	42.65 ± 5.37	54.92 ± 5.55	37.46 ± 8.09	0.03	0.243	0.363	0.002
**Age** (years)	62.39 ± 1.77	60.16 ± 1.38	60.43 ± 1.36	0.552			
**Sex** (M/F)	11/17	10/20	11/19	0.894			

Group 1: Patients without diabetic retinopathy; Group 2: Patients with non-proliferative diabetic retinopathy; Group 3: Patients with proliferative diabetic retinopathy.

One-way ANOVA was used to compare the means of normally distributed data, whereas the Kruskal-Wallis test was used to compare non-normally distributed data.

## DISCUSSION

DR begins with microvascular damage such as the loss of pericytes and continues with ischemia and hypoxia in retinal tissues, ultimately resulting in neovascularization in response to these changes^(19^,^20)^. The pathological process progresses through hypoxia that develops in the ocular tissues^(21^,^22)^. The production of various angiogenic growth factors involved in pathological neovascularization, such as VEGF, insulin growth factor, cytokines, and proteins, is induced by hypoxia^(23^,^24)^.

HIF-1α, the main active subunit of HIF-1, acts as a trans-criptional regulator of critical genes for cellular functions under hypoxia^(9)^. Various studies revealed that HIF-1α protein expression increases as the cellular oxygen level decreases^(25)^. HIF-1α overexpression attributable to abnormal growth and consequent tissue hypoxia has been demonstrated in various tumors^(26)^. Vitreous HIF-1α and VEGF levels increase in parallel in patients with proliferative DR^(27)^. Liu et al. found that HIF-1α expression was higher in ischemic retinas than non-ischemic retinas in their mouse model^(25)^.

Survivin has a unique ability to enhance angiogenesis by inhibiting endothelial cell apoptosis^(12)^. Recent studies illustrated that survivin is rarely expressed in healthy tissues but is usually overexpressed in tumoral tissues such as cancers of the gastrointestinal system, lungs, and breasts^(28^-^30)^.

Because the potential of survivin and HIF-1α to be biomarkers as demonstrated in the aforementioned research, we sought to assess their relationships with the severity of DR. We hypothesized that survivin and HIF-1α levels would increase with ischemia. Therefore, we expected their levels to increase with increasing severity of DR. As we expected, the serum levels of these molecules were higher in patients with non-proliferative retinopathy than patients without levels were lowest in patients with proliferative DR, which features the most severe diabetic involvement. This result can be explained as follows. Panretinal photocoagulation was performed in patients with proliferative DR as a requirement of neovascularization. Because of the ablation of retinal tissues, in which HIF-1α and survivin were released, the levels of these proteins were lowest in patients with proliferative DR. Our findings of higher HIF-1α and survivin levels in patients with non-proliferative DR than in those without DR suggest that the levels of these parameters increase in parallel with the development of DR. Various studies reported that both survivin and HIF-1α levels increase with retinal neovascularization because of intense ischemia. Liu et al. revealed that HIF-1α expression is increased by hypoxia in the early phase of DR^(6)^. In another study, Li et al. reported that HIF-1α expression was increased in the early phase of DR^(31)^. There is further evidence that HIF-1α levels are correlated with the severity of DR^(24^,^27^,^32^,^33)^. Liu et al. confirmed that HIF-1α activates the transcription of survivin during the progression of DR under hypoxic conditions in the ischemic retina. Consequently, overexpression of survivin promotes neovascularization in the retina and DR progression^(6)^.

It might be useful for clinicians to use the results of this study to determine the risk of retinopathy in patients with diabetes and quantitatively evaluate retinal ischemia using serum HIF-1α and survivin levels. In patients with poor blood glucose regulation, ophthalmologists’ prior knowledge of the extent and severity of retinal ischemia could be meaningful for both the physician and patient to implement urgent measures before retinopathy develops or worsens.

This study had several limitations. First, HIF-1α and survivin levels were measured in serum. Measurement of the vitreous levels of these proteins would increase the reliability of the results. The limited indication for vitrectomy in patients with non-proliferative DR makes it difficult to obtain vitreous samples. The major limitation of the study was its retrospective, cross-sectional design. The proliferative DR group did not consist of naïve patients. Because of retinal ablation therapy via argon laser photocoagulation, HIF-1α and survivin expression was not possible in the damaged retinal tissues. In addition, the levels of these proteins were not measured at the time of diagnosis before diabetic retinal involvement, precluding comparisons of the results in this cohort after the development of diabetic retinal involvement. A comparison of these data among the patients and with a control group would provide the most accurate and rational results.

In conclusion, the present study illustrated that serum HIF-1α and survivin levels are higher in patients with non-proliferative DR than in those without DR. Further prospective studies with long-term follow-up in naïve patients could contribute to the literature.

## Authors’ contrıbutıon:

Substantial contribution to conception and design: Burak Bilgin, Semsettin Bilak. Acquisition of data: Semsettin Bilak, Yusuf Özay. Analysis and interpretation of data: Burak Bilgin, Semsettin Bilak. Drafting of the manuscript: Burak Bilgin. Critical revision of the manuscript for important intellectual content: Burak Bilgin, Semsettin Bilak. Have given final approval of the submitted manuscript: Burak Bilgin, Semsettin Bilak, Yusuf Özay. Statistical analysis: Semsettin Bilak. Administrative, technical, or material support supervision: Burak Bilgin, Semsettin Bilak, Yusuf Özay. Research group leadership: Burak Bilgin.
